# Role of Cholecystectomy in Choledocholithiasis Patients Underwent Endoscopic Retrograde Cholangiopancreatography

**DOI:** 10.1038/s41598-018-38428-z

**Published:** 2019-02-18

**Authors:** Chi-Chih Wang, Ming-Chang Tsai, Yao-Tung Wang, Tzu-Wei Yang, Hsuan-Yi Chen, Wen-Wei Sung, Shih-Ming Huang, Ming-Hseng Tseng, Chun-Che Lin

**Affiliations:** 1 Institute of Medicine, Chung Shan Medical University, Taichung, Taiwan; 2 School of Medicine, Chung Shan Medical University, Taichung, Taiwan; 3 Department of Medical Informatics, Chung Shan Medical University, Taichung, Taiwan; 4 Division of Gastroenterology and Hepatology, Department of Internal Medicine, Chung Shan Medical University Hospital, Taichung, Taiwan; 5 Division of Pulmonary Medicine, Department of Internal Medicine, Chung Shan Medical University Hospital, Taichung, Taiwan; 6 Department of Urology, Chung Shan Medical University Hospital, Taichung, Taiwan; 7 Information Technology Office, Chung Shan Medical University Hospital, Taichung, Taiwan; 8 Institute and Department of Biological Science and Technology, National Chiao Tung University, Hsinchu, Taiwan

## Abstract

There are no clinical guidelines for the timing of cholecystectomy (CCY) after performing therapeutic endoscopic retrograde cholangiopancreatography (ERCP) for choledocholithiasis. We tried to analyze the clinical practice patterns, medical expenses, and subsequent outcomes between the early CCY, delayed CCY, and no CCY groups of patients. 1827 choledocholithiasis patients who underwent therapeutic ERCP were selected from the nationwide population databases of two million random samples. These patients were further divided into early CCY, delayed CCY, and no CCY performed. In our analysis, 1440 (78.8%) of the 1827 patients did not undergo CCY within 60 days of therapeutic ERCP, and only 239 (13.1%) patients underwent CCY during their index admission. The proportion of laparoscopic CCY increased from 37.2% to 73.6% in the delayed CCY group. There were no significant differences (p = 0.934) between recurrent biliary event (RBE) rates with or without early CCY within 60 days of ERCP. RBE event-free survival rates were significantly different in the early CCY (85.04%), delayed CCY (89.54%), and no CCY (64.45%) groups within 360 days of ERCP. The method of delayed CCY can reduce subsequent RBEs and increase the proportion of laparoscopic CCY with similar medical expenses to early CCY in Taiwan’s general practice environment.

## Introduction

Cholelithiasis is one of the most troublesome diseases and constitutes a substantial burden on healthcare resources worldwide^1–3^. It requires surgical intervention^4^ and has been seen at an alarming rate over the past two decades in many places, including Taiwan^5^. The abundant access to food in developing and developed countries places the population at increased risk of obesity, and the incidence rates of cholelithiasis grow accordingly^6,7^. Choledocholithiasis results mostly from gallstones passing through the cystic duct into the common bile duct (CBD). Therefore, cholecystectomy (CCY) seems a reasonable method for reducing recurrent biliary events (RBEs) after therapeutic endoscopic retrograde cholangiopancreatography (ERCP) stone removal is performed.

Previous retrospective studies have shown that CCY can reduce RBEs^8,9^ compared with leaving the gallbladder *in situ* after therapeutic ERCP for choledocholithiasis patients. Two prospective small studies demonstrated that CCY has a protective effect for subsequent RBEs for cholelithiasis patients undergoing ERCP^10,11^. Although remnant CBD stones sometimes occurred after CCY^12^, CCY does have the benefit of reducing RBEs. There is no consensus regarding the timing for elective CCY after therapeutic ERCP for choledocholithiasis, despite well-designed retrospective or prospective studies showing 15–20% decrease in RBE rates^8,9,13^ when performing CCY right after ERCP rather than 7–8 weeks later, since some RBEs happened while awaiting delayed CCY. Financial analysis revealed different results for medical expenses for elective CCY after therapeutic ERCP for choledocholithiasis in Western and Eastern countries^9,14^.

There are no current guidelines for the optimal timing for performing CCY after therapeutic ERCP. Few or no real-world studies had proven these theories until a large-scale retrospective database study was performed using an American commercial database^9^. Although this study showed that early CCY can reduce RBEs, and CCY itself can reduce further RBEs in the following year compared with no CCY, the clinical practices in Taiwan are very different due to the different cultural and economic conditions and public health care policies. Therefore, we conducted this current study based on the Taiwan National Health Insurance Research Database (NHIRD).

## Methods

This study was approved by the Institutional Review Board (IRB) of Chung Shan Medical University Hospital, Taiwan. The IRB waived the need for informed consent for this retrospective study based on NHIRD. All methods were performed in accordance with the relevant guidelines and regulations and under surveillance by then IRB of Chung Shan Medical University Hospital.

### Study design

This study is a population-based retrospective cohort study based on Taiwan’s NHIRD, which covered more than 99% of the entire population^15^. The NHIRD has been described in detail in previous studies^16,17^.

**Figure 1:**
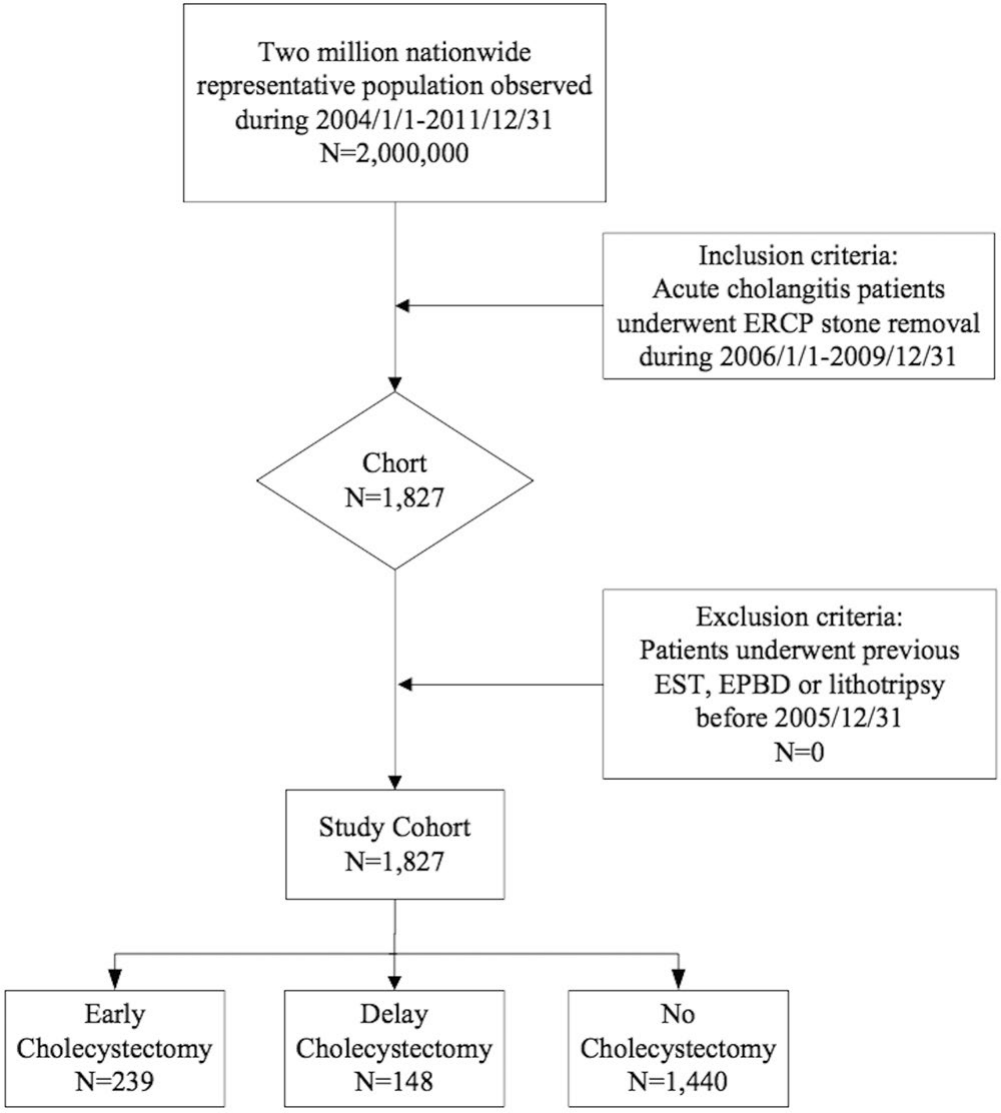
Case selection flow chart of patient selection from two million nationally representative patients in the Taiwan National Health Insurance Research Database. EST: endoscopic sphincterotomy; EPBD: endoscopic papillary balloon dilatation; ERCP: endoscopic retrograde cholangiopancreatography.

Choledocholithiasis cases were selected from two million random samples from the NHIRD between 2004 and 2011 using the Codes of International Statistical Classification of Diseases and Related Health Problems-9th Edition (ICD-9) recorded during admission. Acute cholangitis or choledocholithiasis patients without concurrent cholecystitis were selected using ICD-9 574.2, 574.5, 574.9, 576.1, 576.2 and therapeutic ERCP defined as endoscopic sphincterotomy (EST; order code 56031B, 56033B), endoscopic papillary balloon dilatation (EPBD; order code 56032B) or endoscopic lithotripsy (order code 28008B, 28035B) during index admission occurring in 2006–2009. Patients who previously underwent therapeutic ERCP for choledocholithiasis or CCY, tracing back from index admission to 2004, were excluded. The observation period selected was from January 2010 to December 2011. A total of 1827 patients with symptomatic choledocholithiasis who underwent therapeutic ERCP for stone removal were selected. We further divided these patients into three categories: (1) CCY done at index admission (early CCY), (2) CCY done within 60 days after index admission (delayed CCY), and (3) no CCY done within 60 days after index admission (no CCY). The details of this study design are shown in Fig. 1. Table 1 shows the age, gender, Charlson Comorbidity Index (CCI) score, hospital classification, economic status and living area condition of the total study population. The proportion of laparoscopic CCY and open CCY, RBEs, CCI scores and economic costs were compared for these three groups. RBEs in our study are defined as admissions or emergency room (ER) visits with a diagnosis of cholelithiasis, choledocholithiasis, cholecystitis, cholangitis and pancreatitis. Selective admissions for delayed CCY within 60 days after index admission were excluded from RBE analysis because they were for selected surgery attempts without symptoms. Since there are no outpatient surgery interventions of the biliary system in Taiwan, the financial analysis of admission and ER visits are complete in our national health insurance system data. Economic analysis of costs of index admission, delayed CCY, RBEs visits and total medical expenses were compared for these three groups under Taiwan’s national health insurance system.

**Table 1:** Demographic characteristics of the study population.

Covariate	Data	
	N	SD; %
Age (SD)	65.5	16.2
Male	1018	55.7
Female	809	44.3
CCI score (SD)	3	3.3
Stent	161	8.8
Medical center	1023	56.0
Regional & local hospital	804	44.0
Minimum basic salary (MBS)	942	51.6
1–3 times MBS	767	42.0
Above 3 times MBS	114	6.2
City	1072	58.7
Countryside	725	39.7
Remote village	30	1.6

SD = standard deviation, CCI score = Charlson Comorbidity Index score.

### Data processing and statistical analysis

The National Health Insurance Research Database (NHIRD), with two million subjects who are representative of the nationwide population between 2004 and 2011 in Taiwan, was processed using Microsoft SQL Server 2008 R2 (Microsoft Corporation, Redmond, WA, USA) with the SQL programming language. Statistical analysis was done using OpenEpi: open source epidemiologic statistics for public health, version 3.01^18^. Kaplan-Meier survival analysis was done using R version 3.4.3.

Data obtained from the study were processed using Chi-Square (χ^2^) tests for categorical variables, one-way ANOVA (Analysis of Variance) for continuous variables, and Log Rank (Mantel-Cox) tests for disease-free survival curves. A two-tailed P-value of 0.05 was considered statistically significant in this study.

## Results

The data for 1827 patients who underwent therapeutic ERCP for choledocholithiasis without concurrent cholecystitis were collected. The mean age was 65.5 ± 16.2, and 55.7% were male. In Taiwan, the medical evaluation systems are divided into medical centers, regional hospitals, and local hospitals. Fifty-six percent of these patients received medical treatment at medical centers, with 8.8% receiving endoscopic retrograde biliary drainage stent at index admission; 58.7% of the patients were living in cities. Other detailed information of the study population is shown in Table 1.

Reflecting the culture in Taiwan, surgical intervention was always the last choice for treatment. In our analysis, 1440 (78.8%) of the 1827 patients did not undergo CCY within 60 days after therapeutic ERCP, and only 239 (13.1%) patients underwent CCY during the index admission. Although previous evidence showed early CCY could lead to an increased rate of laparoscopic CCY^9^, and early laparoscopic CCY could reduce complication rates in acute cholangitis^19^ and biliary pancreatitis patients^20^, our study showed a different pattern in Taiwan. Only 89 (37.2%) of the 239 patients underwent laparoscopic CCY in the early CCY group when acute cholangitis was treated at index admission. The proportion of laparoscopic CCY increased to 73.6% (109 of 148) for the delayed CCY group (Table 2).

**Table 2:** Cholecystectomy status of study population.

Variable	Data	
	N	%
Cholecystectomy status	1827	
Early cholecystectomy	239	13.1
Delayed cholecystectomy	148	8.1
No cholecystectomy	1440	78.8
Proportion laparoscopic cholecystectomy		
Early cholecystectomy	239	
Open cholecystectomy	150	62.8
Laparoscopic cholecystectomy	89	37.2
Delayed cholecystectomy	148	
Open cholecystectomy	39	26.4
Laparoscopic cholecystectomy	109	73.6

### Comorbidity profile

The early CCY, delayed CCY, and no CCY cohorts were compared using age, gender and parameters of CCI scores. The results showed significant differences in patient age, CCI scores, underlying congestive heart failure, chronic pulmonary disease, peptic ulcer disease, mild and moderate to severe liver disease, and malignant disease between these three groups of patients. The oldest patients were found in the no CCY group followed by the early CCY group. The proportions of congestive heart failure, chronic pulmonary diseases, and CCI scores were highest in the no CCY group followed by the early CCY group. The proportion of peptic ulcer disease, liver disease, and malignant diseases were highest in the no CCY group followed by the delayed CCY group. The general conditions were the worst in the no CCY patients in our real-world study. All of the CCI score comparison results are shown in Table 3.

**Table 3:** CCI score comparisons between early, delay and no CCY groups.

Covariate	Early CCY 239		Delayed CCY 148		No CCY 1440		p value
	Number	SD; %	Number	SD; %	Number	SD; %	
Age, mean (SD)	63.5	16.7	58.5	17.5	67.1	15.7	<0.001
Female	107	44.8	67	45.3	634	44.0	0.943
CCI score, mean (SD)	2.95	2.88	2.78	2.92	3.91	3.36	<0.001
Myocardial infarction	7	2.9	4	2.7	46	3.2	0.932
CHF	31	13.0	10	6.8	212	14.7	0.026
Peripheral vascular disease	7	2.9	4	2.7	52	3.6	0.758
Cerebrovascular disease	50	20.9	21	14.2	312	21.7	0.104
Dementia	19	7.9	11	7.4	150	10.4	0.291
Chronic pulmonary disease	76	31.8	32	21.6	469	32.6	0.024
Rheumatologic disease	4	1.7	5	3.4	21	1.5	0.216
Peptic ulcer disease	126	52.7	89	60.1	920	63.9	0.004
Mild liver disease	50	20.9	44	29.7	494	34.3	<0.001
Diabetes	69	28.9	39	26.4	487	33.8	0.077
Diabetes with chronic complication	14	5.9	7	4.7	119	8.3	0.162
Hemiplegia or paraplegia	0	0.0	0	0.0	17	1.2	0.100
Renal disease	24	10.0	12	8.1	168	11.7	0.356
Malignancy, including leukemia and lymphoma	33	13.8	21	14.2	318	22.1	0.002
Moderate or severe liver disease	3	1.3	4	2.7	75	5.2	0.013
Metastatic solid tumor	13	5.4	6	4.1	113	7.8	0.123
AIDS	0	0.0	0	0.0	0	0.0	—

CCY = cholecystectomy, SD = standard deviation, CCI score = Charlson Comorbidity Index score, CHF = congestive heart failure, AIDS = acquired immune deficiency syndrome.

### Recurrent biliary events

**Figure 2:**
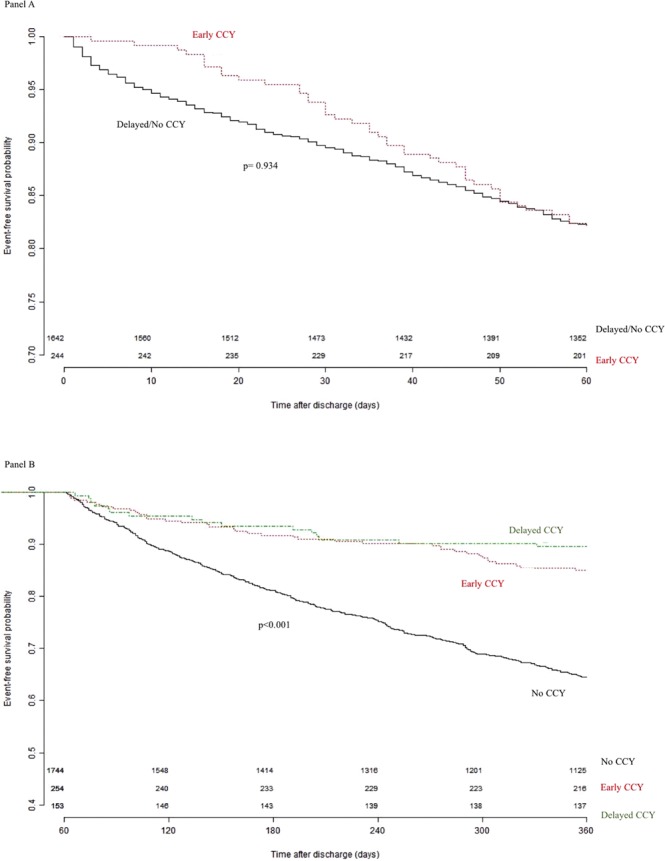
RBE event-free survival curves. (Panel A): Event-free survival curves of early CCY and no/delayed CCY within 60 days after index admission. (Panel B): Event-free survival curves of early, delayed and no CCY groups between 60 and 360 days after index admission.

The analysis of RBEs was divided into two time periods after index admission: from day 1 to day 60, and day 61 to day 360. There were 239 patients who underwent early CCY and another 1588 patients who did not undergo CCY initially at index admission. Within the first 60 days, there were 44 RBEs that occurred in 39 early CCY patients and 292 RBEs that occurred in 238 non-early-CCY patients. The RBE rate was 18.03% in the early CCY group and 17.78% in the non-early-CCY group. There were no significant differences (p = 0.934) between these two RBE rates. RBE event-free survival was analyzed with Kaplan-Meier statistics as shown in Fig. 2A.

During the second time period, from day 61 to day 360, 38 RBEs occurred in 23 (of 239) patients in the early CCY group, 16 RBEs occurred in 11 (of 148) patients in the delayed CCY group, and 620 RBEs occurred in 316 (of 1440) patients in the no CCY group. The RBE event-free survival rates were 85.04%, 89.54%, 64.45%, respectively, and there were significant differences between early and delayed CCY compared to no CCY (p < 0.0001), as shown in Fig. 2B.

### Medical expenses

Medical expenses for index admission, delayed CCY, subsequent admissions and ER visits due to subsequent RBEs, together with total medical charges, were calculated for the three groups. The average medical expense for the early CCY patients at index admission was 146,717 NT dollars, which was significantly higher than that for the delayed CCY and no CCY patients (p < 0.001). The average financial cost for the delayed CCY patients was 81,759 NT dollars. The average cost for RBEs was 11,026 NT dollars for the early CCY group, 8,960 NT dollars for the delayed CCY group, and 28,378 NT dollars for the no CCY group. The average medical expenses for RBEs was the lowest in the delayed CCY group (p = 0.004) and was statistically different from the early and no CCY groups. The average amounts of total medical expenses were the lowest for the no CCY group (p < 0.001) and were similar for the early and delayed CCY groups (p = 0.294). The medical cost comparisons are shown in Table 4.

**Table 4:** The comparisons of medical expenses between early, delay and no CCY groups.

Variable	Index admission		Delayed CCY		Recurrent biliary events		Total charges	
	AVG Expenses (NT $)	p value	AVG Expenses (NT $)	p value	AVG Expenses (NT $)	p value	AVG Expenses (NT $)	p value
Early CCY	146717	ref	0	ref	11026	ref	157743	ref
Delayed CCY	52650	<0.001	81759	<0.001	8960	0.004	143369	0.294
No CCY	77104	<0.001	0		28378	<0.001	105482	<0.001

NT $=New Taiwan dollars, CCY = cholecystectomy, AVG = average, ref = reference.

## Discussion

In our study, the data were collected for patients who underwent therapeutic ERCP for choledocholithiasis without cholecystitis (from a retrospective database that included a random selection of approximately 10% of the Taiwan population), and we noticed that only 21.2% underwent CCY within 60 days of the index admission. This phenomenon can be attributed to cultural factors and the different types of CBD stones (the brownish stones resulting from chronic infection, without gallbladder stones) that play an important role in the Asian population^21^. Forty-four percent of the patients completed treatment at local and regional hospitals (as opposed to medical centers). Compared with current data from Western countries – where the rate of laparoscopic CCY in Australia is well over 95%, the rate of conversion to open CCY is only approximately 2.5%, and the rate of laparoscopic CCY in the United States of America is above 90%^9^ – the proportion of laparoscopic CCY in our results is only 51.16%. The average hospital stay was prolonged in open CCY patients, regardless of whether early or late CCY was performed. This outcome may be another reason that the proportion of patients willing to undergo prophylactic CCY treatment in Taiwan is relatively low.

Regarding the proportion of laparoscopic CCY, we saw a higher laparoscopic CCY rate in the delayed CCY group compared with that of the early CCY group; this outcome was different from the main worldwide study results. This outcome can be explained by noting that (1) we saw more chronic pulmonary disease patients (31.8% vs 21.6%) who were possibly less tolerant of the air effects of the laparoscopic surgery, in our early CCY group; (2) there was a relative low confidence level among surgeons and popularity of laparoscopic surgical equipment in local and regional hospitals in Taiwan between 2004–2011.

### Timing of prophylactic cholecystectomy

Early CCY, rather than delayed CCY (>7 days after admission), is preferable for patients who require hospitalization for acute cholecystitis^22,23^ and selected gallstone pancreatitis patients^24,25^. In the Western world (with better medical standards), the same finding was reproduced for prophylactic CCY for symptomatic choledocholithiasis patients who underwent ERCP. Previous studies showed that early CCY could prevent the RBEs that occurred while waiting for the procedure and reduce the rate of subsequent events. As we know, repeated endoscopic biliary intervention could be used as rescue therapy^26–28^ for dealing with laparoscopic CCY complications. In our study, early CCY did not reduce the interval RBEs compared with delayed CCY for patients of older age and poor cardiopulmonary function. This outcome may have occurred because most clinical studies were done with experienced doctors in well-equipped hospitals. Our results show that early CCY does not guarantee reduction of interval RBEs in a less experienced and less well equipped general practice environment.

We found that CCY, either early or delayed, reduced the subsequent RBEs in the following year; this finding is consistent with the results of many small prospective studies.

### Medical expenses

With financial impact taken into consideration, the method of delayed CCY leads to a smaller medical expense under the clinical practice pattern in Taiwan. The early CCY method leads to an even larger average total medical expense in the following year compared with the no CCY group. After we evaluated the costs of laparoscopic CCY and open CCY, early open CCY patients spent 167115 NT dollars and 19 admission days on average, while late open CCY patients spent 124069 NT dollars and 14 admission days on average. The costs and hospital stays for laparoscopic CCY, 94339 NT dollars in 10 days and 54055 NT dollars in 5 days in early and late laparoscopic CCY patients, respectively, were much lower than that for open CCY patients. The detailed comparisons of laparoscopic CCY and open CCY are listed in the supplementary table. The proportion of open CCY in the early CCY group is high, and this proportion helps to explain why the medical expenses are much higher in the early CCY group compared to the delayed CCY or no CCY groups. Our results support the two-stage treatment clinical pattern^29^ under the current diagnosis-related group (DRG) charges (National Health Insurance) for cholelithiasis used in Taiwan.

There are limitations to our study. For example, medical comorbidity associated with retrospective database analysis-related selection bias may confound the association between the timing of surgeries and the risks of subsequent RBEs. This analysis was conducted using a randomly selected population-based database, reflecting the current medical practice pattern in Taiwan, and we acknowledge the results could be different for highly skilled surgical hospitals. Additionally, the study groups were heterogeneous in age and some parameters of CCI scores. Further prospective randomized studies involving different levels of hospitals may be needed to clarify these issues.

In conclusion, we find that CCY does protect patients from subsequent RBEs in the following year, but early CCY at index admission does not provide protection for choledocholithiasis patients who underwent therapeutic ERCP in general practice in Taiwan. The method of delayed CCY can reduce medical expenses and the risks of subsequent RBEs and increase the proportion of laparoscopic CCY in Taiwan’s general practice environment.

## Supplementary information

Supplementary table
