# Supplementary material for: Role of Cholecystectomy in Choledocholithiasis Patients Underwent Endoscopic Retrograde Cholangiopancreatography
Source: Sci Rep. 2019 Feb 18;9:2168. doi: 10.1038/s41598-018-38428-z (PMC6379409; doi:10.1038/s41598-018-38428-z)
Supplement: Supplementary file 1 — Supplementary table [file 41598_2018_38428_MOESM1_ESM.docx]

**Role of Cholecystectomy in Choledocholithiasis Patients Underwent Endoscopic Retrograde Cholangiopancreatography**

Chi-Chih Wang, Ming-Chang Tsai, Yao-Tung Wang, Tzu-Wei Yang, Hsuan-Yi Chen, Wen-Wei Sung, Shih-Ming Huang, Ming-Hseng Tseng^*^, Chun-Che Lin^*^

**Supplementary table 1** The comparisons of laparoscopic CCY and open CCY

|  | **Early CCY** | | | **Delayed CCY** | | |
| --- | --- | --- | --- | --- | --- | --- |
| **Variable** | AVG Expenses  (NT $) | AVG admission day | AVG Expenses  (NT $) | | AVG admission day |  |
| **Open CCY** | 167115 | 19 | 124069 | | 14 |  |
| **Laparoscopic CCY** | 94339 | 10 | 54055 | | 5 |  |

NT $＝New Taiwan dollars, CCY= cholecystectomy, AVG= average

**Supplementary table 2** RBEs in choledocholithiasis patients with/without GB stone between early, delay and no CCY groups

| Covariate  Repeated ICD-9 coding | Early CCY  239 | | Delayed CCY  148 | | No CCY  1440 | |
| --- | --- | --- | --- | --- | --- | --- |
|  | 39 | | 41 | | 397 | |
|  | With GB stones | Without GB stone | With GB stones | Without GB stone | With GB stones | Without GB stone |
| Case number at index admission | 85 | 154 | 107 | 41 | 751 | 689 |
| RBEs within 60 days | 17 | 26 | 35 | 12 | 123 | 120 |
| RBEs 60-360 days | 17 | 21 | 14 | 2 | 333 | 286 |

CCY= cholecystectomy, GB= gallbladder, RBEs= recurrent biliary events

With GB stones: 574.2, 574.9

Without GB stone: 574.5, 576.1, 576.2

With both ICD-9 574.2, 574.9 or 574.5, 576.1, 576.2; we regard repeated ICD-9 coding as positive for GB stones
